# 2021 Acknowledgment of *mSystems Ad Hoc* Reviewers

**DOI:** 10.1128/msystems.01403-21

**Published:** 2021-12-21

**Authors:** Jack A. Gilbert

**Affiliations:** a University of California, San Diego, San Diego, California, USA

## EDITORIAL

Last year, I hoped that we were moving toward the end of the pandemic. I was wrong. Despite the availability of free vaccines that have demonstrated remarkable ability to reduce transmission and serious illness, vaccine rates are still woefully low. It seems that many of our fellow global citizens (that have access to vaccines) have rejected the evidence presented, preferring to put themselves, family members, and the wider community at risk of serious illness and death. However, this apparent rejection of science should not reduce our resolve to provide rigorous review of our peers’ work. Scientific discovery works only because we as a community are willing to assess its accuracy and validity. Providing this service free of charge is an important part of reducing perceived bias. That being said, it is a major time commitment, and every one of us that gives up our limited time to support peer review does so with the understanding that our assessments are important in ensuring that unfounded, nonrigorous results and conclusions are not disseminated widely. The suggestion that we should abandon peer review and let postpublication review sort through the rigor of new science does not account for the new world in which we live. People will use “published” research to justify their perspectives, and so the propagation of poorly designed studies and inaccurate findings will further the erosion of the public’s “faith” in science. Peer review has never been more important than it is now, and the *mSystems* editors (who are also authors and reviewers) thank you for supporting peer review.

Ruth Chingcuangco Abanador
Abdirahman I. Abdi
Lisa Abernathy-Close
Hamada A. Aboubakr
Jônatas S. Abrahão
Zaky Adam
Beth M. Adamowicz
Ameeta K. Agarwal
Surya D. Aggarwal
Daniel Aguirre de Carcer
Mir Alvee Ahmed
Caio Augusto Martins Aires
Samuel L. Aitken
Ashok A. Aiyar
Kola Ajuwon
Robert A. Akins
Md. Tauqeer Alam
Begum Alaybeyoglu
Luis D. Alcaraz
Gajender Aleti
Gladys Alexandre
Nahid Ali
Shaukat Ali
Daniela S. Aliaga Goltsman
Joannie M. Allaire
Jonathan Allen
Rey Custer Allen
Alexandre Almeida
Hassan M. Al-Tameemi
Eva Alvarez
Stefano Amalfitano
Aman Aman Khan
Katherine R. Amato
Alongkorn Amnuaykanjanasin
Amitesh Anand
Cheryl P. Andam
Annette Carola Anderson
Michael Anderson
Rika Anderson
Simon C. Andrews
S. Andreas Angermayr
Nana Y. D. Ankrah
Alessio Aprile
Elizabeth Archie
Farnoosh Arfaee
Héctor Arguello
José M. Argüello
Gunjan Arora
Melinda Marie Ashcroft
Alexander Askenov
Contreras Asuncion
Haruyuki Atomi
Jennifer M. Auchtung
Selcan Aydin
Frank O. Aylward
Taj Azarian
Reha O. Azizoglu
Paul Babitzke
Giovanni Bacci
Varsha Dave Badal
Brian D. Badgley
Jin-Woo Bae
Jake Bailey
Brett J. Baker
Roberto Balbontín
Regina Lucia Baldini
David A. Baltrus
Marcy J. Balunas
Horia Leonard Banciu
Sreejata Bandopadhyay
Noam Bar
Daniel Barkan
Francisco Barona-Gómez
Rodolphe Barrangou
Douglas H. Bartlett
Nick Barts
Marcelo Bueno Batista
Silvia Beatriz Batista
David L. V. Bauer
Andreas J. Bäumler
Joseph Baur
Matt Bawn
Gwyn A. Beattie
William N. Beavers
Tiago Beites
Georgios N. Belibasakis
Aeriel D. Belk
Amanda Bendia
Jose A. Bengoechea
Maureen Berg
Teresa M. Bergholz
Kathryn Bernard
Aude Bernheim
Anthony Bertagnolli
Matthew Bertin
Elizabeth N. Bess
Sharon Bewick
Oliver K. I. Bezuidt
Mohini Bhattacharya
Giancarlo A. Biagini
Lewis Bingle
Jordan E. Bisanz
Pradeep Bist
Pinaki Biswas
Eran Blacher
Jonathan M. Blackburn
Christopher Blackwood
Jorge Blanco
Ran Blekhman
Mark Blenner
Joseph M. Bliss
Louis-Marie Bobay
Thomas A. Bobik
James Boedicker
Alexandria B. Boehm
Nicholas Andrew Bokulich
Andrea Bonetti
James L. Bono
Batbileg Bor
Erik Borchert
Andrew M. Borman
James Borneman
Thomas C. G. Bosch
Joseph M. Bosilevac
Michael J. Bouchard
Yann F. Boucher
Patrick H. Bradley
Eefjan Breukink
Amanda J. Brinkworth
Shaun R. Brinsmade
Juliana Delatorre Bronzato
Julie Brothwell
Titus Brown
Linda Brubaker
Spencer A. Bruce
Sebastian Bruchmann
Bryan D. Bryson
Gregory A. Buck
Angus Buckling
Silvia Bulgheresi
Lorinda Bullington
Lisa Burdette
Liana T. Burghardt
Mark J. Buttner
Lílian Caesar
Feng Cai
Isabelle Caldelari
Philip Calder
Carole Camarasa
Shawn R. Campagna
Danielle Elizabeth Campbell
Juliana Coutinho Campos
Ahmet Can Berkyurek
Hailong Cao
Jialan Cao
Yueqing Cao
Delphine Capela
Mauricio Caraballo Rodriguez
Valerie Jean Carabetta
Alessandra Carattoli
Juan Pablo Cárdenas
Allison F. Carey
Ross P. Carlson
Ronan K. Carroll
Santiago Castillo-Ramírez
Juan Castro-Severyn
Jorge Cervantes
Kok Gan Chan
Siu H. J. Chan
Trevor C. Charles
Benoit Chassaing
Som S. Chatterjee
Adit Chaudhary
Max Chavarría
Congying Chen
Ding-Qiang Chen
Feng Chen
Gao Chen
Liang Chen
Lianmin Chen
Shaohua Chen
Stanley H. Chen
Yin Chen
Yongliang Chen
Caroline Chénard
Shu Cheng
Alexandra Chiaverini
Jungil Choi
Lon M. Chubiz
Nico J. Claassens
Jan Claesen
Dennis Claessen
Gerard Clarke
Thomas Clavel
Sarah A. Clock
Mara L. C. Cloutier
Shannon R. Coleman
Justine Collier
Eric Collins
Charles Coluzzi
Andre M. Comeau
Fabio Cominelli
Laurie E. Comstock
Terrance G. Cooper
Reilly O. Cooper
Fernando Corrales
Doug Cossar
Lauren Cowley
Katharine Coyte
Sean Crosson
Elena Crotti
David E. Crowley
Nyssa Cullin
Chris Curtin
Roy Curtiss
Leah Cuthbertson
Weronika Czaban
Stefania Daghino
Yang Dai
Benjamin Dainat
Alex Dajkovic
Jennifer L. Dale
Gautam Dantas
Sophie E. Darch
Promi Das
Raunak Kumar Das
Sudip Das
Mark R. Davies
Ellen Decaestecker
Hidde de Jong
Ronnie de Jonge
John P. Dekker
Rosa del Campo
Diego de Mendoza
Xianding Deng
Xiangyu Deng
Xin Deng
Yijie Deng
Laura De Nies
Nicole J. De Nisco
Daniel P. Depledge
Dhwani Desai
Les Dethlefsen
Travis J. De Wolfe
Eric Déziel
Avantika Dhabaria
Neha Dhasmana
Juan Diaz-Colunga
Fabrizio Di Caprio
Gregory J. Dick
Joseph DiDonato
Christian Diener
Stephen P. Diggle
Kimberly Anne Dill-McFarland
Tatiana Dimitriu
Ray Dixon
Ulrich Dobrindt
Yohei Doi
Stephen K. Dolan
Sara Domingues
Justin J. Donato
Sharon Donovan
Charles J. Dorman
Gavin M. Douglas
Marc Drolet
Chao Du
Yabing Duan
Breck A. Duerkop
Seána Duggan
Jack Dunkle
Anne Duplouy
Matt Durrant
Sanjucta Dutta
Rachel J. Dutton
Mária Džunková
William D. Eaton
Tom Edlind
Collin Edwards
Martinique Lefevre Edwards
Marie A. Elliot
Joshua Elmore
Emiley Ansley Eloe-Fadrosh
Melinda Anne Engevik
Christoph Engl
Tobias Engl
Brendan Epstein
Ana V. Espinel-Ingroff
Carmen Espinosa-Gongora
Andreia B. Estrela
Yanhua Fan
Rongxiang Fang
Weiguo Fang
Yufeng Fang
Séamus Fanning
Sara Federici
Victor Fedorenko
Conor Feehily
Fengqin Feng
Hong Feng
Pinghui Feng
Gabriel da Rocha Fernandes
Juan Carlos Gutierrez Fernandez
Marine Feyereisen
Noah Fierer
Reinhard Fischer
Ross Fitzgerald
Kyle Floyd
Fatima Foflonker
Marco Fondi
Herrison Fontana
Gad Frankel
Patrick Frantom
Paul Fraser
Alessandra Frau
Steven Frese
Maximilian Frey
Michael W. Friedrich
Ville Friman
Julia Frunzke
Yunhe Fu
Chikara Furusawa
Giovanni Gallo
Michael G. Ganzle
Cheng Gao
Xiaofeng Gao
David Garcia-Callejas
Melanie Gareau
Junkal Garmendia
Matthew J. Gebert
Jennifer Geddes-Mcalister
John A. Gerlt
Gisa Gerold
Monica L. Gerth
Landon John Getz
Rohit Ghai
Murad Ghanim
Raad Gharaibeh
Partho Ghosh
Scott Michael Gifford
Concha Gil
Micaela Giorgini
Mathieu Gissot
Stefanie P. Glaeser
Anum Glasgow
Laura Glendinning
Erin S. Gloag
Gregory B. Gloor
Alain P. Gobert
Matthew Robert Goddard
Daryl Gohl
Joanna B. Goldberg
Joao Carlos Gomes-Neto
Laura Gómez-Consarnau
Angela Gomez-Simmonds
Yanhai Gong
Cesar Raul Gonzalez-Esquer
Uri Gophna
Tobias Goris
Andrew Gorringe
Jeffrey M. Grabowski
David E. Graham
Todd M. Greco
Chris Greening
Gwen G. Grelet
Joel S. Griffitts
Inês Ramos Grilo
Ghjuvan Grimaud
Ted Grunberg
Alexander Grünberger
Danxia Gu
Haiwei Gu
Steinn Gudmundsson
Roberto M. C. Guedes
Caitriona M. Guinane
Xianwu Guo
Tony Gutierrez
Andras Gyorgy
Maria Hadjifrangiskou
Tatsuro Hagi
Andrea Hahn
Vanessa L. Hale
Alex Hall
Ruth M. Hall
Kambiz Hamadani
Trinity L. Hamilton
Thomas H. Hampton
Mei-Ling Han
Nabil Hanna
Geoffrey D. Hannigan
Pamela Hanson
William R. Harcombe
Jessica Hardin
Christopher Harmer
Xavier Harrison
Mark E. Hart
Christiane Hassenrück
Graham F. Hatfull
Roland Hatzenpichler
Alyse Hawley
Susu He
Yan He
Aziz Heddi
Jussi Heinonsalo
Klaas J. Hellingwerf
Chris Henry
Michael A. Henson
Melissa M. Herbst-Kralovetz
Daniel Philipp Ralf Herlemann
Mark T. Hernandez
Ander Hernández Plágaro
Robert L. Hettich
Paul G. Higgins
Falk Hildebrand
Luke Hillary
Joshua W. K. Ho
Shang-Tse Ho
Ying-Ning Ho
Wouter D. Hoff
Deborah A. Hogan
Devin B. Holman
Michael J. Holmes
Yuichi Hongoh
Matthew Horton
Masahito Hosokawa
Cristina Howard-Varona
Ansel Hsiao
Cheng-Chih Hsu
Jianzhong Hu
Xiaoting Hua
Hsin-Ho Huang
Kerwyn Casey Huang
Shi Huang
Lori B. Huberman
Casey Hubert
Luciano F. Huergo
Diarmaid Hughes
Mylene Hugoni
Amanda Hurley
Alan Hutchison
William P. Inskeep
Richard E. Isaacson
Ralph R. Isberg
Siva Sankari Iyer Mani Sankaran
Robert Jackson
John Jacobs
William R. Jacobs
Myrna Ellen Jacobson Meyers
Deborah Jacobs-Sera
Pratik Jagtap
Nico Jehmlich
Che Ok Jeon
Jong Jeong
Aashish Jha
Juquan Jiang
Yong Jiang
Dong-Yan Jin
Aaron Johnson
James R. Johnson
Michael David Leslie Johnson
Timothy Johnson
Rheinallt M. Jones
Peter Jorth
Christine Josenhans
Laurence Josset
Peter Jungblut
Nadeem O. Kaakoush
Madhavi L. Kakumanu
Marina G. Kalyuzhnaya
Krishna Kantsharma
Heidi B. Kaplan
Smruthi Karthikeyan
Justin R. Kaspar
Laura A. Katz
Emily Kawaler
William L. Kelley
Libusha Kelly
Hans Kestler
Nemat O. Keyhani
Amjad Khan
Nicolas Kieffer
Kristopher Kieft
Young Ho Kim
Jeffrey Alan Kimbrel
Kerry Kinney
Katharina Kitzinger
Maia Kivisaar
Stephanie Kivlin
Jen Knies
Eric Koch
Gerwald Koehler
Arash Komeili
Peter Konturek
Bon-Sang Koo
Omry Koren
Saori Kosono
Ariangela J. Kozik
Frauke Kracke
Elizabeth B. Kujawinski
Anand Kumar
Ashwani Kumar
Roshan Kumar
Anthony Kusalik
Priyanka Kushwaha
Anders Kvarnheden
Man Jae Kwon
Young Min Kwon
Cédric Laczny
Beatriz Lagunas
Leo Lahti
Erh-Min Lai
Iain L. Lamont
Julie LaRoche
Sabina Leanti La Rosa
Peter E. Larsen
Sunil Laxman
Carlito B. Lebrilla
Raphael Ledermann
Chang-Ro Lee
Dong-Woo Lee
Dong-Yup Lee
Eun Yeol Lee
Kihyun Lee
Sunjae Lee
Owen P. Leiser
Vanessa Leone
Keith N. Leppard
Karine G. Le Roch
Cammie F. Lesser
Ka Yin Leung
Mark Alexander Lever
Janina P. Lewis
Bo Li
Fu-Li Li
Huiying Li
Jian Li
Jilian Li
Liang Li
Mu Li
Rongyu Li
Tuo Li
Wen-Jun Li
Xiangzhen Li
Xiaogang Li
Xiaotong Li
Qiming Liang
Josie Libertucci
Tami Lieberman
Jean Lim
Bruno P. Lima
Gipsi Lima-Mendez
Jonathan Y. Lin
Yao-Cheng Lin
Nilton Lincopan
Dan Lindner
Steven E. Lindow
Roger G. Linington
Di Liu
Jinxin Liu
Long Liu
Shirong Liu
Xiaolu Liu
Yan Liu
Yongxin Liu
Yubing Liu
Jason Lloyd-Price
Ted Loch-Temzelides
Andrew Long
Reid Longley
William Lopes
Jose L. Lopez-Ribot
Martina Lori
Wai Yee Low
Catherine Lozupone
Peter Adrian Lund
Elaine Luo
Xin M. Luo
Jonathan Lynch
Meinan Lyu
Zhe Lyu
Bo Ma
Zhonghua Ma
Nataliia Machushynets
Roderick I. Mackie
Hannah MacLeod
Guerrino Macori
Erica L.-W. Majumder
Iris Maldener
Ashish Malik
Johan Malmström
Serena Manara
Julia Manasson
Lauralie Mangeot-Peter
Adriana Mantegazza
Huiling Mao
Costas D. Maranas
Abelardo Margolles
Clarisse (Lisa) Marotz
Philippe Marteau
José Luis Martínez
Augusto Martinez-Antonio
Esteban Martínez-García
Vincent G. Martinson
Takako Masuda
Sébastien Matamoros
Prince Peter Mathai
Miguel A. Matilla
Cameron McBride
Laura-Isobel McCall
Jessica R. McCann
Ryan McClure
Ursula M. McCormack
Ali McCully
Jason E. McDermott
Timothy R. McDermott
Andrew McDowell
Michelle McGuire
Aoife J. McHugh
Michael J. McInerney
James B. McKinlay
Michael J. McLaren
Joel McManus
Rainer U. Meckenstock
Gregory L. Medlock
Maliheh Mehrshad
Luis C. Mejia
Melissa Melby
Silvia Melgar
Brett L. Mellbye
Alessio Mengoni
Guillaume Méric
Noha M. Mesbah
Stéphane Mesnage
Ella M. Meumann
George Michail
Firas S. Midani
Peter E. Midford
Joseph R. Mihaljevic
Jeremiah J. Minich
Biswapriya Biswavas Misra
Rajeev Misra
Richa Misra
Douglas A. Mitchell
Megan C. Mladinich
Jennifer M. Mobberley
Luke A. Moe
Amin Mohamed
Yassene Mohammed
Omkar Satyavan Mohite
Mashkoor Mohsin
Wendy Mok
Lázaro Molina
Denise Monack
Isha Monga
Jonathan M. Monk
Douglas Monteiro
Olimpio Montero
Matthew D. Moore
James J. Moran
Christopher E. Morgan
Megan Morris
Mark Morrison
Jamie Morton
Luis Mota-Bravo
Jochen A. Mueller
Ryan Sean Mueller
Mandy Muller
Catherine Mullié
Alise Muok
Gemma G. Murray
Mario E. Muscarella
Jillian Myers
Carey D. Nadell
Niranjan Nagarajan
Drew R. Nannini
Adrienne B. Narrowe
Payman Nasr
Stephen Nayfach
Daniel Naylor
Brittany Needham
Jeniel E. Nett
Meina Neumann-Schaal
Joshua Patrick Mark Newson
Christopher Nicchitta
Graeme William Nicol
Pierre Nicolas
Vincent Nieto
Pablo Ivan Nikel
Ben Niu
Qiuhong Niu
Franklin Nobrega
Samuel-Philip Nobs
Trent Northen
Spencer V. Nyholm
Anna O'Brien
Conor P. O'Byrne
Kazuhiro Ogai
Dele Ogunremi
Yasuo Ohnishi
Shigeaki Ohno
Naoko Ohtani
Anil Ojha
Andrew Oleinikov
Angela Oliverio
Christine Olson
Marc Ongena
Mei Ooi
Todd Osmundson
Orla O'Sullivan
Hiroshi Otani
Ronan F. O'Toole
Michael Otto
Hong-Yu Ou
Marc Ouellette
Jeremy George Owen
Egon Anderson Ozer
Martin Pabst
Cara T. Pager
Melinda Paholcsek
Sepideh Pakpour
Magnus Palmblad
Robert J. Palmer
Bernhard O. Palsson
Meichen Pan
Tansol Park
John Parkinson
Julio Parra-Flores
Anutthaman Parthasarathy
Laila Pamela Partida-Martinez
Sally R. Partridge
Adrian Paskey
Edoardo Pasolli
Alessandro Passera
Kiran Patil
Michael Patnode
Andrew C. Pawlowski
Jennifer L. Pechal
Itsik Pe'er
Desheng Pei
Rudy Pelicaen
Dale A. Pelletier
Xuanxian Peng
Jakob Pernthaler
James Peters
Scott Peterson
Vanessa V. Phelan
Brett E. Pickett
Giovanni Pilloni
Jarone Pinhassi
Ameet J. Pinto
Johann Pitout
Paul J. Plummer
Mircea Podar
Georg Pohnert
Laurent Poirel
Suchawan Pornsukarom
Anais Potron
Dominic Poulin-Laprade
Akbar Adjie Pratama
Susan Prescott
Erin P. Price
Morgan N. Price
Sambhawa Priya
Alexander J. Probst
Daniele Provenzano
Qiang Pu
Alexandra E. Purdy
Fahd Qadir
Xiaopeng Qi
Zhe-Xue Quan
Alisha Quandt
Kenjiro Wake Quides
Ralf Rabus
Mark Radosevich
Malini Rajan
Mirjana Rajilić-Stojanović
Gordon Ramage
Kelly Ramirez
Dipak Ramji
Jayasimha Rao
David Rasko
Simon Rasmussen
Daniel Rath
Thomas Rattei
Alison Ravenscraft
Kasie Raymann
Timothy D. Read
Shawna Reed
Pierre Renault
Jose M. Requena
Henry Reyer
Alejandro Reyes
Philip J. Richards
Jason M. Ridlon
Daniel Rios Garza
Henrik Munch Roager
Steve Robbins
Leah Roberts
Serina L. Robinson
Amalia Roca
Jennifer Rocca
Guillermo Rodrigo
Marcio Rodrigues
Luis Rodriguez
Eduardo Rodríguez-Román
David Romero
Marilyn J. Roossinck
Jason W. Rosch
Federico Rosconi
Benjamin Ross
Daniel E. Ross
Omar Rossi
Hannes L. Rost
Christopher Rota
Daniel Röth
Simon Roux
Denis Roy
Syamal Roy
Daniel Rozen
Peter Rubbens
Jeffrey Rudolf
Marc Ruitenberg
Steffen Rupp
Fracesco Russo
Sangryeol Ryu
Zakee L. Sabree
Rajib Saha
Jason W. Sahl
Sakuntala Saijai
Elisa Salvetti
Nadia Sampaio
John C. Samuelson
Nicholas Sandoval
Robert A. Sanford
Dominique Sanglard
Alvaro San-Milán
Alyson E. Santoro
Christian Santos-Medellín
Guillaume Sarrabayrouse
Jimmy H. Saw
Tomoo Sawabe
Gary Sawers
Matthew J. Scarborough
Joy Scaria
Joseph Schacherer
Lennart Schada von Borzyskowski
Isabelle Schalk
Dirk-Jan Scheffers
Bernhard H. Schink
Guy Schleyer
Patrick D. Schloss
Marian L. Schmidt
Thomas M. Schmidt
Dirkjan Schokker
Michelle Schorn
Benjamin Luke Schulz
Egbert Schwartz
Hannah Doris Schweitzer
Vera Schwierzeck
Peter Sebo
Leopoldo Segal
Jana Seifert
Ryan F. Seipke
Yuji Sekiguchi
Charlie Seto
Luca Settanni
Emmanuel Severi
Mohammad R. Seyedsayamdost
Lauren Marie Seyler
Michael Shaffer
Maulin P. Shah
Yatrik Shah
Fergus Shanahan
Xihui Shen
Paul Sheridan
Junling Shi
Andrey N. Shkoporov
Amanda Shore
Natalia Shulzhenko
Seyed Davar Siadat
Nikolai Siemens
Susanne Sievers
Cynthia B. Silveira
Amit Singh
Anoop Singh
Baneshwar Singh
Ramandeep Singh
Naresh Singhal
Sonia Singhal
Evan Skowronski
Timofey Skvortsov
Lloyd M. Smith
Melvyn Smith
Paul Smith
Thomas E. Smith
Scott D. Soby
Christian Sohlenkamp
Bokai Song
Hyun-Seob Song
Jiuzhou Song
Justin L. Sonnenburg
Utkarsh Sood
Lauren Speare
Stephen Spiro
Sanjeeva Srivastava
Lee F. Stanish
Fabian Staubach
Bärbel Stecher
Todd R. Steck
Bruno Stefanon
Eike J. Steinig
Ulrich Stelzl
Bo Maxwell Stevens
Bradley S. Stevenson
Adrie J. Steyn
Paul Stodghill
Sarah Strauss
Wolfgang R. Streit
Jian Qiang Su
Xiaoquan Su
Woo Jun Sul
Snorre Sulheim
Chaomin Sun
Hongzhe Sun
Hui-Zeng Sun
Qiang Sun
Shan Sun
Wenxian Sun
Xiaolun Sun
Per Sunnerhagen
Maxim Svetlov
Austin D. Swafford
Bryan Swingle
Jason B. Sylvan
Yinjie J. Tang
Windy Tanner
Gerald W. Tannock
Xuanyu Tao
Azuma Taoka
Ilma Tapio
Cormac Thomas Taylor
Jan Tebben
Herve Tettelin
Christoph A. Thaiss
Andrew Maltez Thomas
Julie A. Thomas
Sunnie R. Thompson
Casper Thorup
Guo-Bao Tian
Xiaojun Tian
Yun Tian
Marius Belmondo Tincho
Kara A. Tinker
Dirk Tischler
Andrzej Tkacz
Jeffery K. Tomberlin
Parizad Torabi-Parizi
Dieter M. Tourlousse
Maxime Tourte
Sachia Jo Traving
Gena D. Tribble
Denis Trubitsyn
Gareth Trubl
Stephen Kwok-Wing Tsui
Jennifer Anne Tullman
Benjamin John Tully
Christine Y. Turenne
Peter J. Turnbaugh
Caroline Turner
Elhanan Tzipilevich
Daniel Udwary
Juan A. Ugalde
Raphael Valdivia
Yo-Ann Valez Justiniano
Ethan Van Arnam
Françoise Van Bambeke
Jan Roelof van der Meer
Karen Jane Vanderwolf
James L. Van Etten
Rob Van Houdt
Jolanda van Leeuwen
Willem Van Schaik
Suzan Pantaroto Vasconcellos
Delyana Peteva Vasileva
Nic M. Vega
Sandeep Venkataram
Vittorio Venturi
N. Verdile
Ajit Vikram
Valeska Villegas-Escobar
Antony T. Vincent
Uwe Voelker
Angelina Volkova
Aurele Vuillemin
Joseph Thomas Wade
Maggie R. Wagner
Irene Wagner-Dobler
Levi Waldron
David Ian Walker
Wolfgang Wanek
Chengshu Wang
Haixia Wang
Jinfeng Wang
Joyce Wang
Junjun Wang
Pandeng Wang
Qiyao Wang
Weilan Wang
Xifeng Wang
Xueshan Wang
Yue Wang
Yongbao Wang
Indu Warrier
Alex D. Washburne
Tilmann Weber
Zhaojun Wei
Virginia Weis
Alexandra J. Weisberg
Jake Weissman
Roy D. Welch
Jia Wen
I. Wheeldon
Nicole Wheeler
Richard Allen White III
Sivaramesh Wigneshweraraj
Roland Conrad Wilhelm
Michael Wilkins
Julia Willett
Amy D. Willis
Tomasz Wilmanski
Jennifer R. Wilson
Jennifer H. Wilson-Welder
Angela Witte
Alan J. Wolfe
Sul Woo Jul
Jonathan Wren
Gerard D. Wright
Robyn J. Wright
Hao Wu
Ruonan Wu
Yu-Wei Wu
Sander Wuyts
Claudia Wylezich
Kristine M. Wylie
Peng Xing
Jinbo Xiong
Wei Xiong
Libin Xu
Ping Xu
Zhenjiang Zech Xu
Katherine Xue
Takuji Yamada
Masayuki Yamamoto
Qingyun Yan
Tao Yan
Xianghua Yan
Baowei Yang
Chunfu Yang
Jinkui Yang
Jun Yang
Zhaomin Yang
Yu-Feng Yao
M.-N. Frances Yap
Robert Yarchoan
Emily Yates-Doerr
Jung-Yong Yeh
Huabing Yin
Shibu Yooseph
Loubna Youssar
Eizadora T. Yu
Guangtao Yu
Jing Yuan
Mengting Maggie Yuan
Carlos R. Zárate-Bladés
Raz Zarivach
Ahmed Zayed
David Zeevi
Anna C. Zemke
Lauren A. Zenewicz
Karsten Zengler
Angela Zhang
Guangliang Zhang
Jiachao Zhang
Jian Zhang
Mingzi Zhang
Pei Zhang
Rong Zhang
Ruifu Zhang
Xiao-Hua Zhang
Yongjun Zhang
Zhengguang Zhang
Kun Zhao
Yingming Zhao
Jun Zheng
Jusheng Zheng
Shijun J. Zheng
Yong Zheng
Haokui Zhou
Jianhua Zhou
Zhi-Gang Zhou
Baoli Zhu
Wenhan Zhu
Yang Zhu
Yong-Zhang Zhu
Ryan M. Ziels
Nadine Ziemert
Michael Zimmermann
Zhiyong Zong
Jackie Zorz
Jasenka Zubcevic
Martin Zwanzig

